# Biological Nanopores: Engineering on Demand

**DOI:** 10.3390/life11010027

**Published:** 2021-01-05

**Authors:** Ana Crnković, Marija Srnko, Gregor Anderluh

**Affiliations:** National Institute of Chemistry, Hajdrihova 19, 1000 Ljubljana, Slovenia; marija.srnko@ki.si (M.S.); gregor.anderluh@ki.si (G.A.)

**Keywords:** nanopores, pore-forming toxins, sensing, aptamers, oligomerization

## Abstract

Nanopore-based sensing is a powerful technique for the detection of diverse organic and inorganic molecules, long-read sequencing of nucleic acids, and single-molecule analyses of enzymatic reactions. Selected from natural sources, protein-based nanopores enable rapid, label-free detection of analytes. Furthermore, these proteins are easy to produce, form pores with defined sizes, and can be easily manipulated with standard molecular biology techniques. The range of possible analytes can be extended by using externally added adapter molecules. Here, we provide an overview of current nanopore applications with a focus on engineering strategies and solutions.

## 1. Introduction

Nanopore-based sensing is an emerging technology with great potential for the detection of diverse organic molecules, sequencing of nucleic acids, and single-molecule analyses of enzymatic reactions and protein folding. Conceptually, nanopore biosensing belongs to the so-called resistive-pulse methods. A classic example of such a method is the Coulter counter, originally developed in the 1950s to count blood cells [1]. The instrument contains a capillary, which is divided into two parts by a wall containing a 20 µm–2 mm aperture. The capillary is filled with an electrolyte solution and an applied electric field causes ions to move through the opening, creating a constant ionic current. As the blood cells move through the narrow aperture, they partially block the aperture, causing a decrease in solution conductivity. This decrease in conductivity (also called resistive pulse) occurs at a frequency equal to the cell number, while its amplitude depends on the cell size. As with other resistive-pulse methods, the particles to be analyzed must have a diameter close to the diameter of the aperture. Thus, the size of the aperture dictates the detection limit. Using the Coulter counter, particles with a size of ~10 μm could be detected [1]. An increase in the detection limit came in the 1970s when it was shown that particles ~100 nm in size could be detected using nuclear-track etched pores with a diameter of 500 nm [2]. While the sensitivity of this device was sufficient to detect particles as small as viruses [3], increasing the limit enough to detect molecules requires molecule-sized apertures, such as those found in natural membrane channels and pores.

The use of biological nanopores for resistive-pulse detection of molecular analytes was made possible by developments in the field of ion-channel physiology in the 1960s and 1970s, namely the development of planar bilayer recording [4] and the development of single-channel current measurements [5]. However, endeavors to combine the principles of the resistive-pulse method with single-channel measurements, thus laying the foundation for nanopore biosensing were not made until the late 1980s [6]. Neutral polymers, originally used to investigate the volume of an ion channel [7], were the first to be recognized as potential analytes [8]. Early studies used channels generated by alamethicin, an antibiotic peptide [8], *Staphylococcus aureus* α-hemolysin, and cholera toxins [9,10]. Possibly the biggest push for the development of nanopore biosensing came after it was found that single-stranded DNA and RNA could be threaded through a nanopore, making it a potential technology for nucleic acid sequencing [11]. Since then, the number of applications for nanopore biosensing has grown immensely, along with improvements in instrumentation and data processing methods.

**Figure 1:**
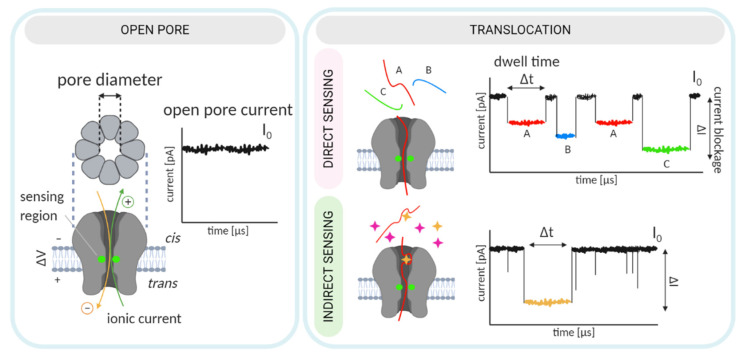
An illustration of a biological nanopore and the principle of nanopore-based biosensing. Direct sensing relies on the distinct current signatures produced by the translocation of individual analytes (top right). Indirect sensing employs an additional, adapter molecule that specifically recognizes the analyte (bottom right). The adapter (shown in red) specifically interacts with the analyte (yellow) and the translocation of the adapter•analyte complex results in a unique current signal.

Nanopore biosensing is based on naturally occurring protein pores. In a typical experiment, the pores are embedded in a lipid bilayer, which separates two chambers, *cis* and *trans*, filled with electrolyte solution (Figure 1). An applied voltage causes ions to move through the pore and create an electrical field. An analyte can be captured and transported across the pore by different mechanisms [12]. For charged analytes, electrophoresis may be the dominant form of transport, carrying the analyte toward the electrode of opposite polarity [13]. For neutral or less-charged molecules, electroosmotic flow may be the dominant force directing capture and/or transport [14,15]. Furthermore, an analyte of appropriate size can enter the pore and alter the ionic current.

An analyte may change the ionic current by (i) producing a change in the electric field within the pore [16] or through (ii) volume exclusion and binding of ions to the traversing analyte, which reduce the ionic current [17]. Importantly, because numerous ions accompany the passage of a single analyte, a large electrical amplification occurs during a single-molecule translocation [18]. In a simplified scenario, the duration and amplitude of the current alteration is unique to each analyte or monomeric unit, in the case of polymer analytes (Figure 1).

Nanopore-based sensing can employ both naturally occurring, protein pores and manufactured, solid-state nanopores [19]. Furthermore, nanopores composed of DNA origami [20] as well as hybrid pores that combine several types of pores [21,22,23] are also being developed. Specific advantages may be found for all systems. Due to their durability over a wide range of conditions and relative ease of integration with microfluidic devices, certain applications can only be realized through the use of solid-state nanopores (e.g., [24,25]). However, biological nanopores, although not as robust as solid-state nanopores, offer better reproducibility due to their defined channel sizes [26]. In addition, lipid bilayers exhibit much lower electrical noise than the materials used to manufacture solid-state nanopores [27].

The repertoire of biological pores is vast. In nature, various nanometer-sized pores act as ion channels, porins, aquaporins, pore-forming toxins (PFTs), and viral pores. Most often, however, nanopore technology applications employ PFTs involved in immune or infection mechanisms [28]. One of the major advantages is that PFTs are expressed as soluble monomers; oligomerization and pore formation occur spontaneously upon contact with the membrane of the host [29,30,31]. The expression of PFT monomers in *Escherichia coli* enables a straightforward production of the precursor, which is subsequently induced for nanopore formation (i.e., oligomerization) using reconstituted lipid surfaces with defined lipid content (either vesicles or bilayers) [32,33].

For sensing purposes, nanopore technology generally exploits two distinct concepts: (i) direct sensing (in which changes in the current arise as the analyte traverses and directly interacts with the nanopore [34]), and (ii) indirect sensing (in which translocation of an adapter molecule that specifically interacts with the analyte is monitored; Figure 1, [35]). The selected sensing approach directs engineering efforts, which can consequently be oriented toward pore mutagenesis or modification of the adapter molecule. These two strategies are described in detail in the following sections. In this review, we provide a summary of recent nanopore applications with a focus on engineering strategies and solutions critical for both direct and indirect sensing approaches.

## 2. Features of the Most Frequently Employed Natural Nanopores

What makes a protein pore a good candidate for biosensing purposes? Considering nature’s own arsenal, engineering efforts can be directed toward pore-forming toxins [36], viral pores (e.g., [37]), porins and aquaporins (e.g., [38]), and even nuclear pore complexes [39]. Properties such as pore diameter, the distribution of charges on the pore’s inner surface, pore stability, and ease of production (i.e., solubility and tagging) are crucial (Box 1). Furthermore, the lengths of the transmembrane channel and its sensing region as well as the geometry of any associated vestibular regions will directly influence the applicability of the naturally occurring pore.

Box 1 **Desired properties of a protein nanopore.**

- Ease of manufacturing;
- Superior stability
- Robust upon engineering (via mutagenesis, insertions, or deletions);
- Uniform or flexible oligomerization (depends on application);
- Narrow constriction(s) and a short channel;
- Low noise in conductance measurements;
- Absence of gating;
- Facile insertion into model membrane bilayers or artificial hydrophobic supports;
- A large raio between open-pore and blocked-pore ionic currents.

Nanopore sensors derived from PFTs, such as α-hemolysin (α-HL) or cytolysin A (ClyA), are typically expressed in *E. coli* as soluble monomers and isolated using affinity chromatography or other means of conventional chromatography (e.g., [40]). Other channel types, such as *Mycobacterium smegmatis* porin A (MspA), may require isolation from the native source [41]. Following purification, isolated PFT monomers can be used in conductance measurements directly or after induced pore formation, which is typically performed with the aid of artificial liposomes. Thus, PFTs that can easily form pores directly on the bilayer system used for measurements may be more convenient for application purposes. In this respect, β-barrel proteins may be a more suitable choice, as they insert into lipid membranes more readily than those that possess an α-helical transmembrane region.

Adequate pore performance in sensing experiments is characterized by discernable current readings obtained from the passage of each molecule or monomer, in the case of polynucleotides and peptides. In other words, the translocation of each molecule is accompanied by current changes that are characteristic in amplitude, duration (dwell time), and frequency (Figure 1). General pore topology, especially its internal geometry and the distribution of charged amino acids in the pore lumen, has a major influence on the pore’s sensing capabilities [42]. Structurally, an ideal pore should have a short and narrow constriction within its channel so that the ionic current blockades originate from only a couple of monomers traversing the narrow constriction (i.e., the pore’s sensing region) at a given point in time.

**Figure 2:**
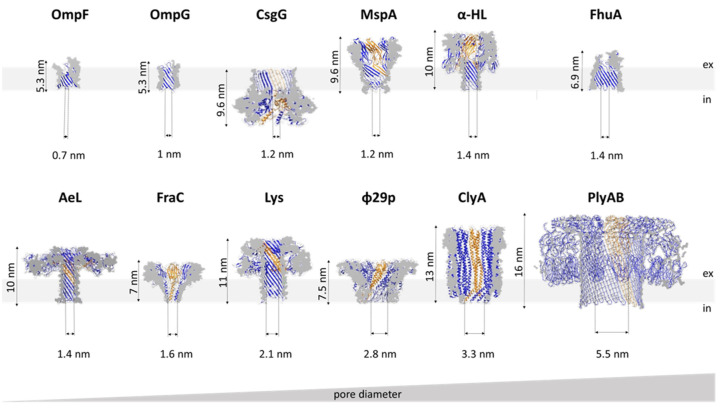
Cross-sections and overall dimensions of the most frequently used protein nanopores. “Ex” and “in” denote the extracellular and intracellular sides, respectively. Individual monomers are highlighted in yellow. Dimension are given for the total length of the pore and the narrowest part of the transmembrane channel (i.e., the sensing region). Outer membrane protein F (OmpF): a single β-barrel of the *E. coli* outer membrane protein F (PDB ID 2ZFG), OmpG: *E. coli* outer membrane protein G (PDB ID 2JQY), CsgG: *E. coli* curli transport channel CsgG (PDB ID 4UV3), MspA: *M. smegmatis* porin A (PDB ID 1UUN), α-HL: *S. aureus* α-hemolysin (PDB ID 7AHL), FhuA: *E. coli* ferric hydroxamate uptake component A (PDB ID 1BY3), AeL: *A. hydrophila* aerolysin (PDB ID 5JZH), FraC: *A. fragacea* fragaceatoxin C (PDB ID 4TSY), Lys: *Eisenia fetida* lysenin (PDB ID 5EC5), φ29p: φ29 phage DNA packaging motor (PDB ID 1FOU), ClyA: *S. enterica* cytolysin A (PDB ID 6MRU), PlyAB: *Pleurotus ostreatus* pleurotolysin (PDB ID 4V2T).

Examination of the electrostatic potential and open vs. blocked pore ionic currents of the most widely used pores, e.g., α-HL, MspA, *E. coli* curli transport channel CsgG, and aerolysin (AeL), delineates how geometry and charge distribution influence pore performance [43]. Although these four pores differ in their lumen geometry, they all possess a narrow sensing region with a diameter of ~1 nm (Figure 2). In MspA, the short constriction is positioned at the intracellular entrance of a funnel-shaped channel. In CsgG, the constriction is located approximately in the middle of an hourglass-shaped channel. In α-HL, the constriction is positioned toward the extracellular part, separating the broad vestibule from the transmembrane channel. The cylindrical β-barrel channel of α-HL is relatively narrow (2.4 nm). For this reason, the current is influenced by a larger number of monomers (nucleotides) than in the cases of CsgG and MspA. Similar to α-HL, AeL possesses a cylindrical lumen shaped by the β-barrel; however, it has two constrictions at the intracellular and extracellular parts, respectively. Compared to the other three pores, AeL contains more positive charges in its lumen.

Nevertheless, electrostatic potentials drastically drop at the sensing regions of all pores. However, in α-HL and AeL, the drop appears to be caused by the presence of charged amino acids, whereas in MspA and CsgG, which possess neutral amino acids, the drop appears to be directed by the pore’s geometry. MspA and CsgG exhibit the highest open-pore currents, likely due to the combination of a short constriction length and broad channel. Conversely, the narrow β-barrel channels of α-HL and AeL result in relatively low open-pore currents. However, since their respective blocked pore currents follow the same pattern, their respective current reductions (ΔI, Figure 1) are almost identical, indicating that all four pores represent good candidates for sensing [43].

### 2.1. Pores Containing β-Barrel vs. α-Helical Transmembrane Region

Ideally, a single protein nanopore could be easily adapted for the detection of chemically and structurally diverse analytes. While various adapter molecules can aid detection in an indirect manner (see below), direct sensing may still be preferred, as it requires preparation of the protein component only. As direct detection is most sensitive when the analyte and pore diameter are similar in size, one of the most sought-after features is the ability to modulate pore size [34].

To modulate pore inner diameter, bulkier amino acids can be incorporated at the sensing region, or the entire oligomer structure can be redesigned. The latter is possible with pores possessing an α-helical organization of the transmembrane section, as these pores naturally show higher flexibility in oligomer assembly [44,45]. Experimental conditions, such as pH or mutating the residues involved in lipid interactions, may enable capturing nonstandard oligomer assemblies that are desired for a particular application [46]. However, variable pore shapes and sizes can result in multi-level signals rendering such pores unsuitable for detection purposes or requiring additional engineering [47]. In contrast, β-barrel assemblies may be resistant to changes in oligomer structure, but their robust pore formation typically results in clear, step-like signals [48].

For both pore types, the successful identification of analytes relies on the width and amino acid composition of the narrowest region of the pore, the sensing region [6] (Figure 1). This is especially true for polymers that are composed of different building blocks, such as DNA, RNA, and proteins. As nanopore-based sequencing employs conductance measurements during macromolecule translocation, and the electric field is more intense at the narrowest section of the pore, the signal is dominated by the residues occupying the space in the constriction and not the total length of the channel [49]. For this reason, engineering of the sensing region can be sufficient for analyte detection, precluding the need to drastically alter the structure of the pore oligomer.

### 2.2. Most Frequently Employed Pores: β-Barrel Proteins

β-barrel protein channels located in the outer membrane of Gram-negative bacteria and some Gram-positive mycobacteria represent a class of highly desirable pores due to their extreme resistance to thermal and chemical denaturation [50]. General porins, such as the outer membrane protein G (OmpG) or MspA, form water-filled pores that allow the passage of relatively small hydrophilic substances, according to their concentration gradient. Active transporters, such as the iron-siderophore receptor FhuA, are also interesting targets for biosensing applications (Table 1).

MspA, the major porin of *M. smegmatis*, forms a homo-octameric pore with a transmembrane region composed of antiparallel β-strands [51] (Figure 2). It is one of the most stable porins characterized to date, in terms of both subunit dissociation and monomer unfolding [50]. The pore structure is retained over a wide range of pH values (2–14) and temperatures (up to 92 °C) as well as in the presence of detergent [50]. In this respect, MspA surpasses the stability of porins from Gram-negative bacteria and is thus attractive for biotechnological purposes. The MspA channel is ~10 nm long and has a funnel shape that is ~5 nm wide on its extracellular side. However, a single narrow constriction at the opposite end has a diameter of only 1 nm and quickly transitions to a much wider area. This shape of the sensing region makes it well suited for DNA detection, for which it was adapted to early on [41]. As indicated by the potential of mean force analysis, a large energy barrier at the constriction of wild type (wt) MspA exists for all four DNA nucleotides. In the M2 mutant (MspA D90N/D91N/D93N/D118R), commonly used for DNA sequencing, this barrier disappears with the removal of the negative charges from the narrow constriction [38,52]. A structurally similar porin from *Nocardia farcinica* has been used to investigate the translocation of cationic peptides [53] and cationic cyclodextrins (CDs) [54]. Its channel is cation-selective, but in contrast to MspA, it is composed of two distinct subunits (NfpA and NfpB).

**Figure 3:**
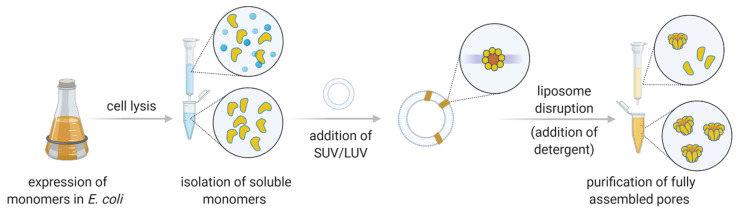
A generalized laboratory pipeline for the production of protein pores from pore forming toxins. Following expression in *E. coli*, the soluble monomers (shown in yellow) are isolated via chromatography. Addition of small or large unilamellar vesicles (SUV or LUV, respectively) induces spontaneous oligomerization and pore formation (a top view of a theoretical octamer is shown). Upon disruption of liposomes by addition of detergent, the mature pores are purified to remove unreacted monomers and improperly assembled pores.

The use of monomeric β-barrels may facilitate targeting individual positions in the pore and thus the ease of purification (Figure 3). Specifically, corresponding efforts using homo-oligomeric pores require precise oligomerization and purification of the target variants. One such protein is OmpG from *E. coli*. The structural analysis of OmpG revealed a 14-stranded β-barrel architecture [55]. On the extracellular side, β-strands connect to each other by long loops, while on the intracellular side, they are bridged by short turns. At neutral pH, the protein adopts an open conformation but closes at acidic pH. In the latter state, one of the β-strands partially unfolds, and the loop that is generated by this motion folds back into the pore’s lumen, thereby occluding the pore [55]. This structural information is in agreement with conductance measurements, which have shown that wt OmpG undergoes pH-dependent, voltage-dependent, and spontaneous switching from the open to the closed state, i.e., gating [56]. Wild type OmpG closes at pH < 7 and at voltages >±100 mV. Furthermore, at neutral pH and voltages <±100 mV, the pore gates spontaneously. To eliminate this behavior, the mobility of one of the loops must be decreased and the hydrogen bonding pattern between strands must be increased [57,58]. Interestingly, although gating is generally regarded as undesirable behavior, wt OmpG has been used for protein detection, observed as alterations in the gating patterns resulting from changes in the mobility of loop 6 [59].

Another monomeric β-barrel membrane protein, the ferric hydroxamate uptake component A (FhuA), transports ferrichrome-bound iron across the outer membrane of *E. coli*. The structure of the transporter shows an antiparallel β-barrel with a central channel blocked by a plug, the cork domain [60]. The wt protein is >700 amino acids long and possesses 22 anti-parallel β-strands that shape a ~7 nm long pore [60]. On the intracellular side, the β-strands are connected by 10 short β turns, while on the extracellular side, much longer loops connect the strands. To adapt the protein for biosensing purposes, considerable redesign is necessary. For instance, deletions of the cork domain and large extracellular loops are required for conductance experiments [61]. Curiously, the addition of an N-terminal hexahistidine tag increases the already large unitary conductance from ~4 to ~8 nS in 1 M KCl [62].

### 2.3. Most Frequently Employed Pores: Pore-Forming Toxins

Pore forming toxins encompass a diverse group of amphipathic proteins from a wide range of organisms. PFTs exhibit diverse structures and are typically classified according to the secondary structure of the transmembrane region (Table 1). Produced as soluble monomers, PFTs oligomerize upon interaction with the target membrane and undergo structural rearrangements that allow them to form mature transmembrane pores (Figure 3; [29,30,31,63]). The purpose of PFT secretion depends on the organism: bacteria may use them as virulence factors, while eukaryotes usually employ PFT as part of a defense mechanism [64]. The transmembrane region of PFTs may be shaped as a β-barrel or an α-helical structure, and can thus be divided into two groups, i.e., α-PFTs and β-PFTs.

α-HL is a PFT (leukotoxin) secreted by *S. aureus*. The pore, an obligatory heptamer [48], spans the membrane with a β-barrel channel and has a conical vestibule on the extracellular side (Figure 2). The pioneering work by Menestrina revealed that α-HL displays gating activity, albeit with a much lower frequency than that of natural ion channels. Importantly, the addition of divalent and trivalent cations increased the gating frequency [65]. Later analyses showed that gating activity increases at higher applied potentials but decreases at higher pH or at higher concentrations of monovalent salts in the recording buffer [36,66]. Narrowing down the conditions that ultimately abolished α-HL gating enabled the use of α-HL in sensing applications. The seminal work of Kasianowicz et al. demonstrated that ssDNA and RNA can traverse the α-HL pore under applied potential and that the duration of observed blockades depends on the length of the nucleic acid chain [11]. These experiments paved the way for the nanopore-based DNA sequencing used today. In addition to ssDNA [67,68,69,70,71] and RNA [72,73,74], α-HL has been used to detect DNA hairpins [75], peptides [76,77], proteins [78], and various other analytes (see below).

AeL, a heptameric β-PFT from *Aeromonas hydrophila*, is secreted as an inactive precursor that is activated through proteolytic cleavage of the C-terminally placed loop by proteases located at the surface of the host cell [79,80]. The pore formed by mature AeL monomers exhibits a unique fold, with two concentric β-barrels stabilized by hydrophobic interactions [81]. While this double barrel fold endows the AeL nanopore with high stability, the narrowness of its inner lumen affords well-separated current signals and dwell times that enable reliable signal interpretation. The short and wide vestibular region is further responsible for low-noise open currents. Due to this high sensitivity, AeL has been used for the detection of DNA [82,83], peptides [77,84,85], proteins [86], and individual amino acids [87]. AeL can distinguish individual nucleobases [88], modified cytosines in serum samples [89], oligonucleotides of different lengths [83], and mixed homopeptides [84]. The transport of unfolded proteins can also be studied by AeL [86]. Another aerolysin-type nanopore suitable for biosensing is lysenin, which has been engineered for DNA detection through the removal of five negative charges from its transmembrane region [90]. Single-channel currents show that the mutations facilitate translocation of a DNA aptamer, but also reduce the noise at positive applied potentials.

### 2.4. Most Frequently Employed Pores: α-Helical Pore-Forming Toxins

ClyA is an α-helical PFT with a comparatively large diameter (3–6 nm, depending on the entrance). To form the dodecameric pore, individual protomers must undergo substantial rearrangements that involve approximately half of their residues [91]. Due to its wide inner lumen, the pore is a suitable candidate for the detection of medium-sized proteins, which might enter the lumen of the ClyA pore. Both *E. coli* and *Salmonella enterica* serovar Typhi harbor highly similar ClyA homologs; however, in conductance measurements, *S. enterica* ClyA is more desirable because of its lower noise [92]. *S. enterica* ClyA has a signal-to-noise ratio 10-fold higher than that of α- HL or MspA, although only within a certain range of the applied potential [93]. Interestingly, the exact oligomer structure of *E. coli* ClyA is controversial: the crystal structure shows a dodecamer [91], whereas cryo-electron microscopy structures show 8 [44] or 12–14 subunits [94]. Owing to its size, ClyA has been successfully used to capture proteins and study their conformation [95], investigate binding configurations of a protein•DNA complex [96], and observe dsDNA translocation at high (2.5 M, [97]) and physiological salt conditions [98].

Fragaceatoxin C (FraC) belongs to the actinoporin family of PFTs. These α-helical PFTs form pores in the presence of sphingomyelin [29,99,100,101]. Conformational changes in the N-terminal part of the protomer are responsible for the transformation of the protomer from the water-soluble to the membrane-traversing state [99]. The pore has a fairly narrow transmembrane region that connects to a wider vestibule on the extracellular side. The diameter of the narrow constriction is 1.2 nm. Although this is sufficiently wide for ssDNA to thread through, mutations are necessary to achieve passage of DNA strands. Mutation D10R, necessary to allow DNA translocation, needs to be accompanied with another, distal mutation that repairs low hemolytic activity of said variant (K159E). Double D10R/K159E FraC variant (ReFraC) has been used to resolve homopolymeric polynucleotides [102]. FraC has been used to resolve proteins with sizes between 1.3 and 25 kDa [103]. The combination of mutagenesis of the membrane-binding residues and electrolyte pH shows that FraC has the flexibility to form hexameric and heptameric pores in addition to octamers that preferentially form from wt monomers at neutral pH [46].

In addition to the most commonly used porins and PFTs listed so far, other pores with promising properties for sensing have been investigated. One example is the transport channel CsgG. Its mutated version (the so-called R9 series) with improved signal quality is used in MinIONs—small portable DNA sequencers used by Oxford Nanopore Technologies [104,105,106]. The pore is a 36-stranded β-barrel with a 4 nm wide channel in which the narrow constriction is centrally located (Figure 2). Recent work has shown that the natural ligand CsgF improves the sequencing accuracy of medium-sized homopolymeric tracts [107]. The dodecameric φ29 DNA phage packaging motor has a 3.6 nm wide channel through which dsDNA passes during packaging and infection (Figure 2). This may make it suitable for the detection of large analytes, such as double-stranded DNA [37] and proteins [108]. It was also adapted to detect small molecules, such as ethane, thymine, and benzene [109]. Another viral protein, the thermophage G20c portal protein, can be inserted into lipid bilayers when conjugated with porphyrin [110]. Other potentially useful pores include pleurotolysin (PlyAB, [111]) and the outer membrane protein F (OmpF; [112]), which have been explored for the detection of proteins and ampicillin, respectively (Figure 2).

## 3. Direct Sensing Using Nanopores: Engineering upon Demand

### 3.1. Inherent Requirements of the Method

**Figure 4:**
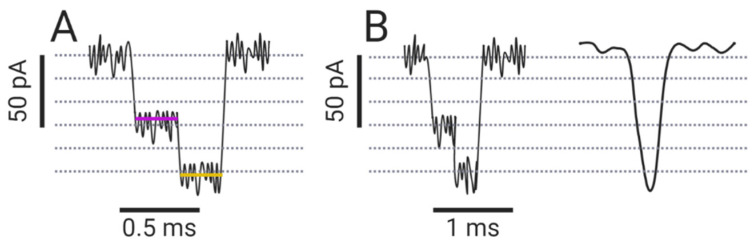
(**A**) A schematic representation of a multiple-level blockade. The smaller amplitude is highlighted in purple, the larger in gold. (**B**) Loss of information due to over-filtering.

Arguably, no sensing is possible without appropriate analysis of the electrical signals. In a typical experiment, up to 200 mV is applied across the pore, resulting in currents in the pA to low nA range. The current signal is collected and amplified by the amplifier and directed to the digitizer, which converts this analog data into digital data. The recording and sampling rates of the amplifier and digitizer are in the low µs range, which sets the limit for maximum temporal resolution [113]. An analyte can cause a single- or multiple-level blockade, i.e., a blockade with different amplitudes (Figure 4A). Each current blockade is characterized by its amplitude, dwell time, and interevent intervals, which are collected and plotted as histograms. Gaussian fits are used to determine the amplitudes of the blocked pore current and the most probable dwell time of the analyte, while the exponential fit is used to determine the mean interevent interval. For a detailed description of the processing of the collected data for nucleic acids and peptides, we refer the reader to references [114] and [115], respectively.

One of the fundamental problems in data processing from nanopore experiments is to distinguish the changes in ionic current caused by the analytes from the statistical oscillations of the signal (i.e., noise) generated by the electronics and the nanopore system itself. Since amplitude resolution is primarily limited by noise, low-pass filtering is applied, which improves signal-to-noise ratio (SNR), but lowers temporal resolution (Figure 4B). Low-pass filters, which remove the signal above a certain frequency (cut-off frequency), should be used with caution so as not to over-filter the data, as this can lead to inaccurate determination of the blocked-pore currents (Figure 4B) [116].

A high signal-to-noise ratio also depends on pore properties. Already in the 1990s, it was recognized that noise can arise due to reversible protonation of residues in the channel [16]. A strong pH dependency may indicate that the noise originates from residue ionization, as was demonstrated for α-HL [16] and OmpF [117]. In addition, effects such as gating and channel breathing may influence the noise observed in the system [118]. A detailed examination of the underlying sources of noise in nanopore-based systems confirms that temporal conformational changes and ionizable residues in biological pores significantly contribute to the observed noise [27].

Another universal requirement for nanopores used for sensing applications is the absence of gating. Interestingly, all pores spontaneously switch between their conductive states (i.e., gate). This may render them unusable for biosensing purposes. However, PFTs such as α-HL, may gate much slower than natural Na^+^ or K^+^ channels (by ~4 orders of magnitude) [36]. Thus, nanopore selection may be directed toward natural pores with a low gating frequency. In other cases where the gating behavior can be attributed to a specific element, as is the case with the OmpG pore (see above), deletion and/or mutagenesis of certain parts must be undertaken to stabilize the pore in a single conductive state [25,26].

Specific pore requirements are defined by the analyte; in other words, the application dictates the choice of pore. In this respect, the size of the channel is of the utmost importance. As the pore acts as a molecular sieve, the diameter of its channel determines which analytes can pass through. Thus, different pores are suitable for the analysis of different analytes (Table 1, Figure 2). Curiously, in some cases, an analyte that exceeds the pore dimensions can still enter the pore lumen [102,119].

While β-barrel pores can be resistant to changes in the stoichiometry of their oligomers [48], variations in subunit numbers can be fairly permissive for α-helical pores. As mentioned above, mutating residues involved in lipid interactions may facilitate the formation of less stable oligomer structures [46]. Some wt α-helical pores spontaneously assemble into oligomers with a varying number of subunits, which increases the range of targets that can be detected with these pores [119]. In this respect, pores containing an α-helical transmembrane region may be preferred candidates for *de novo* design [120,121].

Amino acids lining the channel walls play a decisive role in nanopore biosensing, as they define its geometry and charge distribution. A recent study provides an excellent example of this. An integrated computational and experimental approach revealed the molecular determinants for the ion conductance, ion selectivity, and translocation properties of the AeL pore [122]. A previous report demonstrated that a pair of residues at the pore’s entrance (R282 and R220) and exit (K238 and K242) act as two main sensing regions due to their steric and electrostatic properties [123]. Interestingly, these steric and electrostatic features appear to be intertwined in their effect. Variants harboring wider pores (e.g., AeL R220A) actually show lower open-pore currents at positive applied potentials, likely due to the decreased positive charge at the pore’s entrance. A similar reduction is observed for R282A variants as well. Conversely, charge removal from the residues at the pore exit does not appear to exert the same effect, indicating that the cap region controls the open pore current. Thus, these residues may be important to achieve suitable current amplitudes with the analyte of choice. However, the dwell time of the translocating molecule correlates strongly with the narrow constriction diameter, which indicates that geometric requirements must also be taken into account [122].

### 3.2. The Use of Accessory Proteins

**Figure 5:**
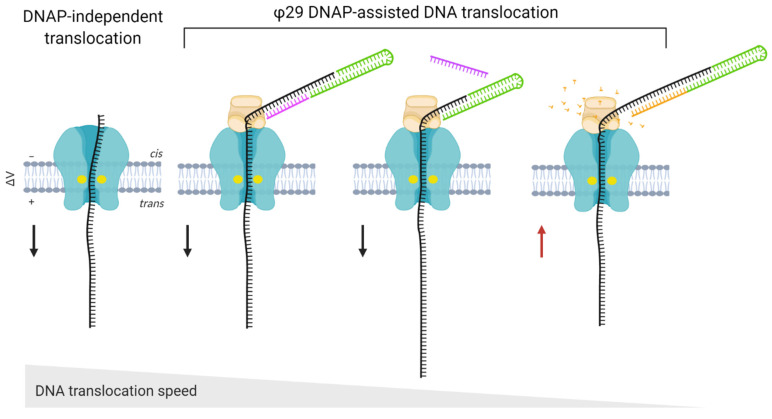
Controlling the speed of DNA translocation with a φ29 DNA polymerase (DNAP). When analyzed alone, the DNA to be sequenced (in black) translocates too rapidly for individual bases to be resolved (left). To allow ratcheting by φ29 DNA polymerase (shown in tan), a primer (green fragment) and a blocking oligomer (pink fragment) are required. The blocking oligomer prevents the φ29 polymerase-dependent extension before it reaches the nanopore. The free 5′ end of the polymerase•DNA complex is drawn toward the pore until the polymerase reaches the entrance of the pore (penultimate left). The applied voltage threads the DNA through the pore, causing the blocking oligomer to dissociate (penultimate right). This exposes the 3′ end of the primer and allows synthesis of the complementary strand (shown in yellow, right). The arrows indicate the direction of DNA movement. The position of the narrow constriction is indicated with yellow dots within the nanopore (shown in cyan). Adapted after [125].

In some cases, additional proteins must be employed for accurate biosensing. For example, ssDNA translocation through α-HL and MspA nanopores occurs at >1 nt/μs, which is too fast to be detected by ionic current measurements [41,124]. The most successful approach to slow down DNA translocation is to pair the nanopore with an enzyme that displaces the DNA in steps that are temporally well-separated and even in duration. A DNA polymerase [125] or a helicase [126] can ratchet the DNA in this way (Figure 5). The choice of the ratcheting enzyme is extremely important for accurate DNA sequencing, as almost half of the errors in sequencing reads are due to its malfunction. This is because the enzyme can accelerate at certain sequences, and can even go backward, returning to earlier positions in the DNA strand [126,127].

The ratcheting enzyme must tolerate high salt conditions and operate at room temperature with high processivity, since the ionic current measurements normally occur at room temperature and in media with high salt content [128]. DNA polymerases from *E. coli* (Klenow fragment) and T7 and φ29 polymerases have all been applied to function as molecular motors to dictate the movement of DNA in an ordered fashion through α-HL [129,130,131]. However, the φ29 enzyme can synthesize very long stretches of DNA under a wide range of forces, which is necessary to efficiently oppose the electrophoretic force required to move the DNA strand through the pore [132]. Furthermore, φ29 DNA polymerase and Hel308 helicase can operate at salt concentrations that are too high for other DNA ratcheting enzymes, making them suitable for use in nanopore-based applications [133].

## 4. Direct Sensing Using Nanopores: Applications

### 4.1. Detection of Nucleic Acids

DNA sequencing using MinION devices is nowadays an established platform. Since the first evidence that nucleic acids can translocate through the α-HL pore [11], this technology has become the main technique for long-read sequencing. Some major events in DNA sequencing using nanopores include the elimination of α-HL gating [16], discrimination between individual homopolymers [74], and detection of individual nucleotides [70]. The importance of DNA immobilization further highlighted the need for a ratcheting enzyme that would slow down ssDNA translocation to the extent that individual nucleobases could be resolved. The φ29 DNA polymerase was shown to fulfill this purpose effectively, as demonstrated by single-nucleotide resolution using α-HL and MspA pores [125,131]. Owing to its long channel (5 nm), α-HL was replaced by MspA, whose 1.2 nm wide and ~0.5 nm long constriction offered better resolution of current signatures obtained for different nucleotides (Figure 2). However, to adapt MspA for the translocation of single-stranded nucleic acids, mutations at its narrow constriction and in other regions had to be introduced, as described above [41].

Other nanopores have also been shown to allow DNA translocation (e.g., AeL, Re-FraC, and lysenin [83,90,102]); however, the vast majority of work has employed α-HL, MspA, and now CsgG pores. Ongoing engineering efforts are mostly directed toward increasing sequencing accuracy. One approach is to further engineer the pores for increased accuracy at difficult regions [107]. Another approach is to use multiple reads to create a consensus sequence, which can lead to very high nucleotide identity (99.5%) [134]. However, the latter is not applicable when the amount of DNA is low as is, for instance, in metagenomic studies. In these cases, accuracy must be achieved at the level of single-DNA passage.

**Figure 6:**
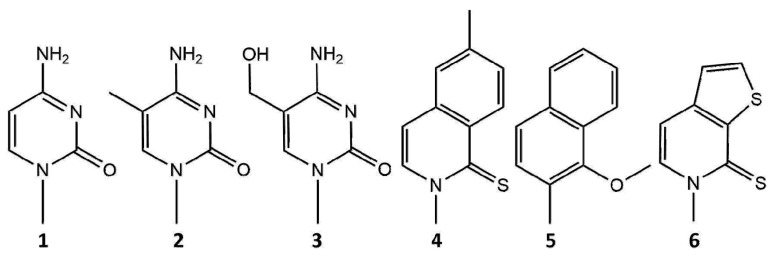
Structures of modified (**2**,**3**) and unnatural (**4**–**6**) nucleotides resolved by nanopore sequencing. **1**: cytosine, **2**: 5-methylcytosine, **3**: 5-hydroxymethylcytosine, **4**: dNaM, **5**: d5SICS, **6**: dTPT3.

Nanopore technology has been increasingly used to detect various nucleotide modifications, including epigenetic markers. Such modifications are biomarkers for various diseases, including cancer. The combination of MspA and φ29 polymerase was used to detect methylated CpG sites [135]. Mapping methylcytosine and 5-hydroxymethylcytosine modifications on DNA is important to gain insights into gene expression. In addition to MspA, detection of these two modifications has also been achieved with α-HL [136] (Figure 6).

Another interesting aspect of nanopore sequencing is the ability to sequence xeno-nucleic acid (XNA)-containing unnatural nucleotides. MspA was used in tandem with φ29 polymerase to detect the unnatural nucleotides dNaM and d5SICS [137] (Figure 6), which were developed to expand the genetic alphabet to three base pairs [138]. In the same work, the authors showed that the Hel308 helicase can also be used to ratchet DNA with the expanded alphabet through the MspA pore [137]. The MspA pore was also used to sequence 2′-deoxy-2′-fluoroarabinonucleic acid (FANA) polymers, in which 2′-fluoroarabinose replaces ribose [139]. The FANA-containing homopolymers poly(U), poly(C), and poly(A) produce distinct signals when detected with an MspA pore. Similar to ssDNA and ssRNA, the translocation of FANA through the pore is too fast to resolve its sequence. However, the choice of a ratcheting enzyme for FANA is narrow, as backbone-modified XNAs are generally rejected by natural DNA polymerases [140]. However, φ29 DNA polymerase may be employed in this instance as it exhibits reverse transcriptase activity when encountering the FANA template [139]. Recent work has shown that MspA can be used to sequence DNA containing the dTPT3-dNaM unnatural base pairs [141] (Figure 6). CsgG may be another suitable pore for the detection of unnatural nucleotides, as the MinION setup has been shown to distinguish between 11 different thymidine analogs [142].

### 4.2. Detection of Proteins: Basic Requirements

Using the power of nanopore sensing technology for proteomic purposes has been an overarching goal for many years. For direct sequencing with nanopores, protein detection (similar to nucleic acid detection) requires (i) compatibility between the pore diameter and traversing protein analyte, (ii) a lack of repulsion between the protein analyte and pore walls, (iii) convenient control of protein movement through the pore, and (iv) unique current signatures for each building block of the analyzed protein. Ideally, nanopore-mediated proteomics would enable long-read sequencing of entire proteins, as has been achieved for DNA. This would require denaturation of the protein analyte, which may not be compatible with biological nanopores (see below). Furthermore, the time scale on which protein translocation occurs is too fast to resolve individual amino acids. Excellent and detailed reviews on challenges and advances in protein sequencing by nanopores can be found (see references [115,143,144]).

Proteins can be detected in their folded form or as fragmented peptides. The majority of biological pores used to date have a very narrow aperture (~1 nm, Table 1), which prevents the passage of entire proteins in their native form. Pores with broader channels, such as ClyA, FraC, and φ29 packaging motor, have been adapted for protein detection [46,93,108]. ClyA has a diameter of 3.3 nm, with a wider *cis* than *trans* opening (Figure 2). Human and bovine thrombin can both be electrophoretically captured within wt ClyA. Although relatively similar, these two proteins produce distinct blockade patterns [93]. Further investigation into ClyA assembly revealed that the wt protein oligomerizes with some flexibility, as indicated by the broad unitary conductance of the preassembled pores [119]. Furthermore, mutating one or both cysteine residues influences the oligomerization process, leading to an increased preference toward larger pores when using a ClyA variant lacking one of the cysteines (ClyA-CS). Adjusting the pore size further dictates the manner by which analytes are captured in its lumen. Capturing human thrombin, which has two residence sites within wt ClyA, reveals preferential binding in the deeper site of residence when using larger ClyA-CS pores. Interestingly, human thrombin not only enters the lumen, but is also translocated, as evidenced by the altered blockade durations, which decrease exponentially with applied voltage. This change is probably not due to thrombin denaturation, as the protein is stabilized by four disulfide bonds, and the residual blocked pore current remains unchanged at different applied potentials when thrombin resides in the ClyA channel [119].

A similar flexibility in oligomerization was revealed for another α-helical PFT, FraC. Mutations in the part of the protein that forms the lipid interface in the mature pore as well as acidic pH during pore assembly influence FraC oligomerization, with increased occurrences of heptamers and hexamers in addition to canonical octamers [46]. Interestingly, pores with lower open pore conductance also show more pronounced cation selectivity. Using the second smallest pore type, angiotensin peptides I–IV can be readily discriminated when analyzed simultaneously (~100 Da resolution) [46]. Angiotensin A and II, which differ in mass by only 44 Da, were successfully resolved using the smaller FraC oligomer. Additional engineering may still be required to enable the sampling of smaller peptides for which the high speed of translocation is still a challenge [46].

### 4.3. Detection of Proteins: Electrophoresis vs. Electroosmotic Flow

Although electrophoresis can control the translocation of nucleic acids due to their uniform charge, the regulation of protein and peptide movement is somewhat more complex. A correlation between peptide mass and current signal was observed with uniformly charged polyarginine peptides analyzed using AeL and T7 motor protein pores [84,145] and with aromatic amino acid-rich peptides analyzed using an α-HL variant containing additional aromatic side chains in its lumen [146]. In all these cases, the mean dwell time and current blockade amplitude increased with peptide mass. As evidenced by the experiments that employed the aforementioned α-HL variant, enhanced resolution can be achieved by introducing additional binding sites into the pore lumen [146].

**Figure 7:**
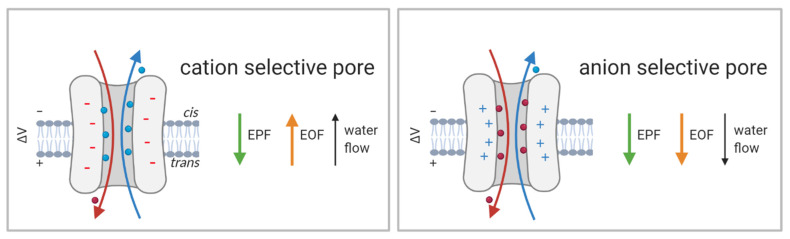
Schematic representation of the electrophoretic force and electroosmotic flow (EPF and EOF, respectively) affecting the motion of a negatively charged analyte in a nanopore experiment. Due to the applied voltage, negatively and positively charged ions (shown as red and blue dots, respectively) move toward the electrode of opposite polarity. The charged residues in the pore lumen simultaneously attract counterions and repel coions, creating an imbalance between the positive and negative charge fluxes, resulting in a net water movement and EOF.

Natural proteins and peptides do not possess a uniform charge distribution; consequently, the electrical field can either facilitate or counteract the transport of certain peptides. Similarly, its contribution to the translocation of peptides containing neutral amino acids may be negligible. Nevertheless, electroosmotic flow (EOF), which occurs in nanometer-sized pores with charged sidewalls, may have a significant impact on the transport of proteins and peptides (Figure 7, [49]). First recognized in experiments with solid-state nanopores [147], EOF may play an important role in translocation through biological pores, despite the fact that biological pores have lower overall charges compared to those of artificial pores. Experiments using α-HL have shown that EOF can drive and unfold small peptides [148] as well as promote their capture at the vestibule entrance [149]. As protein entry into nanopores is directed by passive diffusion, electrophoretic force and/or EOF can significantly influence protein capture. In addition to the shape and charge of the nanopore, its ion selectivity has a major impact on EOF. Experiments with wt FraC and its double variant ReFraC (D10R/K159E) demonstrated that the charges of the amino acids at the narrow constriction (here amino acids at position 10) control the ion selectivity of the entire pore. In this case, a single mutation (D10R) is sufficient to change the ion selectivity from strongly cation- to anion-selective [103]. Consequently, the water flux changes upon mutation of the constriction site residue. While water flows from *cis* to *trans* at negative applied potentials in the wt pore, it does so in ReFraC when positive voltage is applied (Figure 7). Changes in pH, which modulate the protonation state of the constriction site residue, can modulate ion selectivity and EOF and consequently the efficiency of protein capture [103]. Analogous mutations in PlyAB also lead to a change in ion selectivity, impacting the EOF direction. Furthermore, in PlyAB they also cause the appearance of electroosmotic vortices, which are important for protein capture and are already appreciated in solid-state nanopores [111,150].

While it is relatively straightforward to predict which pore residues might prevent nucleic acid passage through charge repulsion, charge alterations may have unpredictable effects on peptide translocation (see above). Because of the intricate interplay between the electroosmotic and electrophoretic transport of proteins and peptides, current efforts appear to be more focused on pH optimization that allows appropriate sampling of protein analytes [103,111,148]. However, the transport of analytes larger than the pore channel may be dictated by electrophoresis, rather than EOF, as demonstrated by the analysis of 540 amino acid-large pertactin passenger domain using the AeL nanopore [151].

### 4.4. Detection of Proteins: Unfolding and Translocation

Many protein analytes are too large to translocate through narrow biological pores in their folded state. One possible solution is to unfold the analyte and thread it through the pore in this state. Interestingly, some biological pores are fairly robust and can remain functional in the presence of elevated denaturant concentrations. This may be especially evident when the pore is already assembled in the lipid bilayer. For instance, α-HL monomers and pores can withstand urea concentrations of up to ~4.7 M and 7.2 M, respectively [152]. The α-HL pores also remain stable in the presence of moderate concentrations of guanidinium (up to 2 M), which is sufficient for the unfolding of a protein analyte [153]. AeL, stable in the presence of 1.6 M guanidinium [86] and at temperatures of up to 70 °C [154], has been exploited to study the translocation of maltose-binding proteins in the presence of guanidinium and their temperature-induced unfolding [86,154,155]. Similar experiments using AeL pores were used to investigate the thermodynamics of guanidinium-denatured pertactin [151]. Alternatively, protein analytes could be unfolded using a motor protein, similar to the ratcheting enzymes used to thread DNA. Compared to thermal or chemical denaturation, enzymatic unfolding could also improve the control over translocation, ensuring processive unidirectional motion of the analyte.

**Figure 8:**
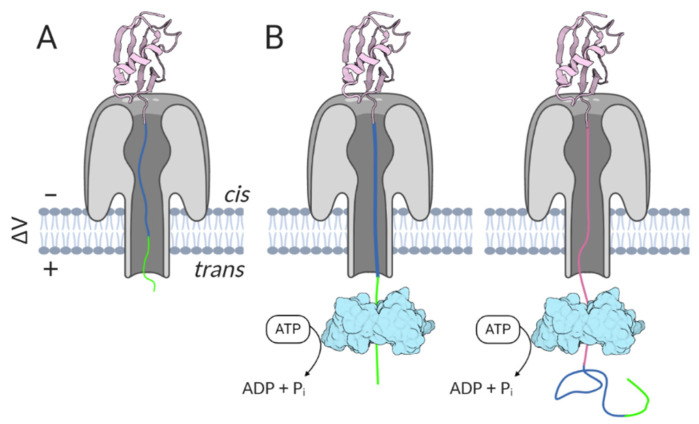
ClpX-mediated unfolding promotes protein translocation through a nanopore. A folded protein analyte (pink) is fused to a charged tag (dark blue), and an ssrA tag (bright green). (**A**) In the absence of ClpX, the charged tag enters the pore lumen; however, the dimensions of the folded protein prevent it from entering the pore. (**B**) When ClpX (shown in cyan) is present in the *trans* chamber, it recognizes the protruding ssrA tag and starts to unfold the analyte in an ATP-dependent manner, thereby ensuring translocation of the entire analyte.

ClpX, an ATPase component of the *E. coli* ClpXP protease, is able to bind, unfold, and translocate several protein targets into the degradation chamber formed by the other protease component [156]. ClpX was used to drive the translocation of ubiquitin-like protein Smt3 variants through the α-HL pore [78]. ClpX was recognized as a suitable enzyme because of its processivity and ability to denature stable proteins. Under applied potential, the Smt3 variant with a C-terminally placed charged flexible linker followed by an ssrA tag starts to move through the pore toward the *trans* side (Figure 8). While the flexible linker and ssrA tag can freely enter the pore, the folded Smt3 domain cannot. However, ClpX present in the *trans* chamber can now access the protruding ssrA tag, and driven by ATP hydrolysis, start to unfold and pull the Smt3 domain through the channel. In the absence of ClpX (or ATP necessary to power the unfolding), stable blockades are observed that are consistent with the folded Smt3 domain being held atop the α-HL vestibule. While ClpX-independent translocation can occur, the events associated with it are longer and less consistent in duration. Importantly, ClpX-assisted translocation appears to be relatively unaffected by the applied potential, as demonstrated by similar analyte dwell times recorded at different voltages [78,157]. A similar strategy has been executed with α-HL and the *E. coli* GroEL chaperone [158].

Ideally, each amino acid of the unfolded protein analyte would be presented at the nanopore’s sensing region and detected by its unique current signal. Using wt AeL, it has been shown that 13/20 natural amino acids, brought to the pore channel using a polyarginine tag, can be discriminated by the sensing region. Blockade currents created by the amino acids Q, M, H, I, and Y are indistinguishable from each other, and the same is true for E and V. The remaining amino acids, however, possess unique current signatures in terms of mean residual current and blockade duration. Residual current is negatively correlated with the excluded volume and the mass of the amino acid. However, a more systematic correlation exists in case of hydrodynamic volume than with molecular mass, as exemplified by the distinguishable current signature produced by the isomeric L and I amino acids [85]. For most of the peptides detected using the differently sized FraC nanopores, the extrapolated volumes obtained for the fully occupied pore indicate volumes that fit the region between residues D10 and D17, and likely define the sensing region space [46]. In α-HL, the constriction region dimensions indicate that it may screen ~1.6 amino acids at a given time. Using peptides designed to be trapped within the pore under applied potential through the stretches of positively and negatively charged residues on their N- and C-termini, it has been shown that α-HL can distinguish between the amino acids A and W [159].

### 4.5. Detection of Post-Translational Modifications and Conformational States of Proteins

One important feature of protein sequencing using nanopores is the potential to detect post-translational modifications. So far, the OmpG pore has been used to indirectly distinguish between glycosylated and unmodified versions of avidin, owing to its negatively charged surface that selectively attracts the glycosylated form of the analyte. Interaction of the pore entrance with the modified analyte manifests in reduced pore current and increased OmpG gating frequency [160]. The ClyA pore has been engineered to detect mono- and polyubiquitinated forms of a medium-sized protein. Using the previously developed ClyA-AS nanopore [119], an additional Q56W mutation was introduced into the pore lumen to increase analyte dwell times; this pore can also distinguish between proteins bearing a ubiquitin modification at different sites [161]. Phosphorylation of entire proteins can be detected by α-HL [162], while AeL and FraC can be used to monitor phosphorylation of model peptides [163,164].

Protein nanopores can be used to examine folding/unfolding pathways [165,166,167] and to probe for the presence of characteristic conformers [168]. The latter requires a pore that is sufficiently large to trap the enzyme in its lumen. To sample conformations of *E. coli* dihydrofolate reductase (DHFR) with ClyA-AS, the analyte is first trapped via its recombinantly added tag and examined in apo- and substrate-bound forms [168]. Rewardingly, this single-molecule examination revealed new conformers and conformational transitions, which escaped earlier analyses of the enzyme [168].

The unique sensitivity illustrated in the last example showcases nanopore sensing as an outstanding label-free technique to examine both functional and structural properties of proteins. However, direct sampling of very subtle changes may require that the protein analyte be trapped within the channel, setting a limit for the size of the analyte. A similar issue arises with protein translocation. To circumvent this problem, many sensing approaches utilize current blockades observed through analyte binding near the pore entrance or partially enclosed within the pore. The flexible choice of the binding ligand allows for highly specific detection of analytes [59], determination of binding constants [108,169,170], and detection of proteins at nanomolar concentrations [108,171]. The translocation-independent approach may thus be the method of choice for the detection of very large proteins for which appropriate pore dimension cannot easily be achieved.

### 4.6. Direct Detection of Other Analytes

Any analyte applicable for biosensing using nanopores must interact with the pore for a sufficiently long time and generate sufficient current blockades to be detected efficiently. In addition, the interaction must be sufficiently specific to enable highly accurate detection. For this reason, small analytes are usually detected via an indirect mechanism (see below). Some interesting examples of direct sensing with analytes other than nucleic acids or proteins include Ga(III) coordination cages of opposing chiralities [172], 2,4,6-trinitrotoluene (TNT) [173], and various mustards using the α-HL pore [174]. To detect TNT, M113 at the narrow constriction of α-HL must be replaced by an aromatic amino acid, such as Y, F, or W [173]. In the case of mustard detection, single or multiple C residues were introduced at position 117 of α-HL and allowed to react; the incremental blocking signal corresponded to the number of bound mustard molecules within the lumen of the pore [174].

Another example of α-HL engineering includes introduction of the Zn(II) binding site via mutagenesis of lumen residues near the trans side entrance [175]. Introduction of histidine at positions 123, 125, 133, and 135 creates a binding site where Zn(II) can be coordinated in the preferred tetrahedral configuration. Upon individual expression of 4H variant and wt α-HL monomers, post-expression pore formation allows the incorporation of a single 4H protomer in the mature heptameric pore (the WT_6_4H_1_ variant pore) bearing only one Zn(II) site. With careful trace editing, the simultaneous detection of Zn(II) and Co(II) was achieved [176].

## 5. Indirect Approaches to Nanopore Sensing

**Figure 9:**
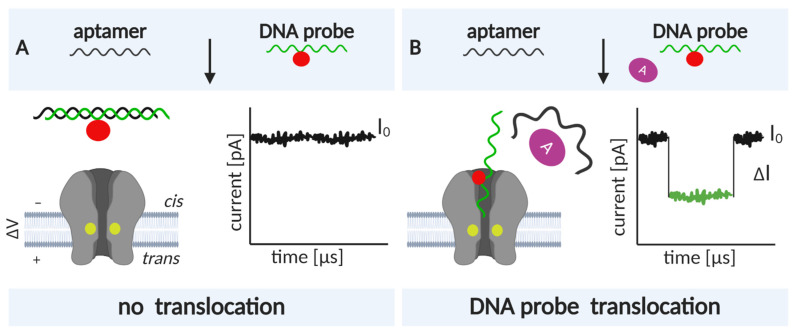
The principle of indirect sensing using an aptamer and a competing, complementary DNA probe. (**A**) In the absence of analyte, the analyte-specific aptamer (in black) forms a duplex with the DNA probe (in green); due to its size, the duplex cannot enter the pore (in grey). (**B**) When the analyte (purple) is added, the aptamer binds the analyte and the free DNA probe can translocate through the pore. The current signature of DNA probe translocation is unique due to labeling and can be distinguished from other signals generated by nucleic acids present in the sample. The position of the DNA label is indicated by a red circle. The sensing region of the pore is indicated by yellow circles.

In this approach, the analyte is detected using an adapter molecule that enables highly specific binding. Consequently, nanopore detection is performed by analyzing distinct current signals arising from (i) analyte•adapter lodging/translocation (Figure 1), (ii) free adapter translocation, or (iii) translocation of a third molecule that competitively binds to the adapter (Figure 9).

**Figure 10:**
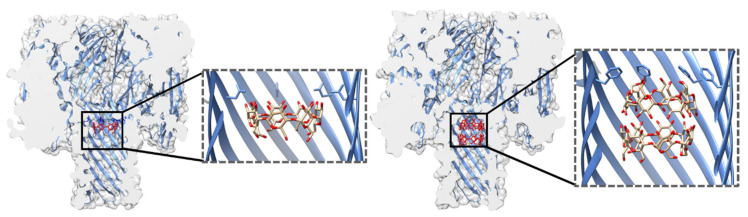
Structures of α-HL variants with, β-cyclodextrin (βCD) adapters reported in [178]. α-hemolysin (α-HL) is shown in blue and βCD in red. PDB IDs: 3M2L (M113F_7_), 3M3R (M113F_7_·βCD), 3M4D (M113N_7_), 3M4E (M113N_7_·βCD).

Early on, β-cyclodextrin (βCD) was recognized as a suitable adapter for α-HL, which can bind it reversibly when added from the *trans* side of the pore. The binding kinetics derived from the current blockades indicate a single binding site within the lumen of α-HL [177]. The binding only partially blocks the α-HL channel, which allows monitoring the interactions of βCD with different target molecules. Examples include adamantane derivatives or pharmaceuticals such as the antidepressant imipramine or the antihistaminic promethazine [177]. Importantly, current signatures may be used for both analyte identification and quantification. If the restriction site M113 is mutated to V, H, Y, D, N, or F, the variant pore binds βCD with 10^3^–10^4^-fold higher affinity than the wt pore [178]. Mutant α-HLs can enable tighter binding of βCD added from the *trans* side (M113F) or from both entrances (M113F/K147N, Figure 10). Such α-HL•βCD complexes can be used for fast examinations of enantiomeric mixtures. The pharmaceutical properties of ibuprofen and thalidomide depend on their enantiomeric state. Distinctly different current signatures can be obtained from the *S-* and *R-*isomers of ibuprofen when βCD is bound to the *cis* or *trans* side of the mutant α-HL pore and the drug is added to the opposite-side chamber. Conversely, a thalidomide mixture was best analyzed when the drug was added to the *trans* chamber and βCD was bound at either *trans* or *cis* side of the pore [179]. However, the βCD adapter may not be suitable for the detection of amino acid enantiomers [180]. Instead, a phenanthroline moiety that coordinates a Cu^2+^ ion is introduced at C117. Amino acids coordinate Cu^2+^ through their amine nitrogen and carboxylic group oxygen, which results in current blockades. Distinct current amplitudes could be observed for racemic mixtures of Y, F, C, and D, with d-enantiomer producing a larger amplitude for all the amino acids except Y [180]. Another CD adapter, heptakis-(6-deoxy-6-amino)-β-cyclodextrin, can be employed with the α-HL M113R variant to examine racemic mixtures of all aromatic amino acids [181].

This α-HL M113R variant was used in combination with a polyamine-conjugated βCD to investigate the kinetics of trypsin cleavage of *N*^α^-benzoyl-l-arginine ethyl ester. This method builds on previous work that used α-HL for trypsin reaction tracking [182,183]. However, in that approach, both the substrate and cleavage products caused current blockades. In the case of polyamine-βCD•α-HL (M113R), only the cleavage product causes current signatures, allowing the determination of steady-state kinetic parameters [184].

While other substituted βCDs [185] and βCD complexes [186] can be used for the purposes of biosensing, adapters, such as cyclic peptides [187], and cucurbiturils [188], can further increase the spectrum of possible analytes. Adapter proteins can be tethered near the *cis* side entrance to selectively capture their protein partners, as demonstrated for the barnase–barstar interaction using the FhuA pore [189,190]. Adapter proteins can be trapped within the pore channel of large ClyA-AS. Through conformational changes of the adapters that accompany analyte binding, concentrations of small molecules, such as glucose or asparagine, can be determined [191]. The ClyA pore was also used to create the protein rotaxane in which the pore itself acts as a macrocycle, *E. coli* DHFR as the first and Strep-Tactin– horseradish peroxidase (HRP) conjugate as the second stopper. The C-terminally placed Strep-tag on DHFR threads through the nanopore channel and binds to the engineered Streptavidin (the HRP conjugate) on the opposite side of the pore. In this state, any diffusion of peptides is prevented but can be enabled by increasing the negative voltage; this allows facile control over protein translocation [192].

Smaller adapter molecules can also be used. Tetrachloroaurate(III) binds to methionine residues near the constriction site of the wt α-HL and MspA-D91M mutant. The bound tetrachloroaurate(III) remains trapped within the MspA channel, enabling the detection of highly similar thiol species, i.e., cysteine, homocysteine, and l-glutathione [193].

### Indirect Sensing: Harnessing the Power of Aptamer Ligands

Due to their highly specific recognition of target molecules and their stability, aptamers may be particularly well suited to act as adapter molecules in nanopore biosensing. Furthermore, their repertoire is likely to grow due to the rapid progress in the selection of these molecules. (Table 2). Initial insights into trapping Y-shaped aptamers into an α-HL pore came from bioinformatic analyses [194]. The first experimental evidence involved trapping G-quadruplexes within the α-HL pore with which the changes in conductance allowed tracking of DNA unfolding and subsequent translocation through the pore [195]. G-quadruplex structures are also a part of thrombin-binding aptamers (TBA). Experiments with the ClyA pore and internalized complexes of human thrombin and TBA revealed two distinct current signatures, demonstrating that TBA binds to thrombin in two orientations [96].

The investigation of DNA/RNA•protein complexes using nanopores may not be straightforward, as the high salt concentration necessary for conductance measurements may cause dissociation of the complex. Conversely, nanopores tend to gate at physiological salt concentrations. Interestingly, the highly truncated FhuA ΔC/Δ4L nanopore is stable at low ion concentrations and was used to inspect the binding of different DNA aptamers to the viral NCp7 protein. As the small NCp7 protein has a significant positive charge, it enters the cation-selective FhuA channel freely, whereas negatively charged DNA aptamers are repelled. By measuring the frequency of current blockades (generated by free NCp7) at different concentrations of DNA aptamers, the dissociation constants for three different aptamers with affinities in the nanomolar and micromolar ranges were determined [212].

MicroRNA, a class of short non-coding RNAs important for cell cycle regulation and various signaling pathways, are known to be difficult to detect in biological samples due to their sub-picomolar concentrations. Using a complementary ssDNA adapter with poly(dC) extensions at both ends and an α- HL nanopore, microRNA was quantified from patient plasma samples. Detection is ensured by the characteristic signature generated by the free poly(dC) tail and the adjacent double-stranded DNA-RNA region [213]. To increase specificity, an analogous strategy using peptide nucleic acid (PNA) adapters was applied. A single nucleotide mismatch in a PNA-RNA hybrid leads to greater energy loss than in DNA-RNA hybrids, making these adapters highly suitable for specific RNA detection [214]. Experiments using an RNA mimic, the locked nucleic acid (LNA), have shown that these types of probes may distinguish between microRNAs differing in only one nucleotide [215]. Consequently, LNA-based biosensing is well suited for the detection of single-nucleotide polymorphisms [216].

Nanopore force spectroscopy has been employed to investigate the binding of aptamers to their targets. This type of spectroscopy is similar to optical tweezers techniques. In short, a folded DNA is trapped within the nanopore, and as the applied voltage increases, so does the electrical force that is applied to the structure, which leads to its unfolding. Once it unfolds, the DNA strand translocates through the pore. This method has been used to study the unzipping kinetics of DNA hairpins by monitoring the rate of unfolding as a function of applied voltage [200,201]. With this method, it is possible to monitor DNA unfolding as it moves from the *cis* to the *trans* chamber [200] or, reversibly, from the *trans* to the *cis* chamber [198,201]. The ability to switch between the two directions is important because, unlike forward translocation, reverse translocation is not significantly affected by pore-DNA interactions [198,201].

Aptamers composed of ssDNA are convenient because in the absence of a ligand, they may unfold and pass through the narrow constriction of α-HL or other pores with a diameter of ~1 nm. However, when the ligand is present, the DNA analyte complex is too large to pass through the pore and is instead captured. The currents corresponding to the translocation and capture are markedly different. Kawano et al. designed a cocaine-binding aptamer (CBA) containing a long poly(dC) tail that facilitates capture and observation of complex binding [210]. The CBA is a Y-shaped structure, which, without the tail, easily escapes from the channel. The tail was designed to occupy the channel in its entirety; previous experiments have shown that a minimum of 25 nucleotides is needed to span the β-barrel to the entrance of α-HL [217]. The fact that DNA and RNA homopolymers create different current amplitudes must be considered when creating tails [218]. The signature blockade levels of poly(dC) tail ensures that CBA is detected with specificity. In the absence of cocaine, blockades characteristic of ssDNA translocation are observed. With the addition of cocaine, complexes are formed, and CBA•cocaine is captured and observed as a long current blockade. The complex can be released by reversing the applied voltage. However, spontaneous openings were also observed, possibly due to DNA unzipping. Measurements with aminobenztropine (a cocaine analog) revealed that the system is highly specific. Further improvements to the sensitivity of this system have been achieved with the introduction of a complementary DNA strand, which competes with the intramolecular binding of CBA [209]. This method relies on the characteristic current signature generated by the translocation of complementary DNA (denoted as the DNA probe in Figure 9). Conversely, CBA, introduced into the *cis* chamber prehybridized to complementary DNA, can only dissociate upon the addition of cocaine, with which it forms a complex. Thus, CBA never passes through the pore. Furthermore, the complementary DNA translocates only if it is displaced from the duplex by cocaine binding; hence, the number of events corresponding to its passage correlate with the number of cocaine molecules in the system. The detection limit in the first system that did not employ the competing, complementary DNA was ~1 µM, close to its theoretical limit [210]. Using complementary DNA, cocaine concentrations as low as 50 nM could be detected from human serum and saliva samples [209].

A similar approach, in which a complementary DNA strand obstructs aptamer•analyte complex formation, was utilized to selectively recognize platelet-derived growth factor B-chain homodimers (PDGF-BB). Here, the aptamer is split into two parts, both containing structured DNA and a long single-stranded region. The single-stranded regions anneal to each other when brought into proximity by PDGF-BB binding. In the absence of PDGF, one of the split aptamer DNAs hybridizes with the complementary ssDNA probe. Similar to the system for cocaine detection, the ssDNA probe is released from the complex with the aptamer in a quantitative manner, and the number of events corresponding to its translocation matches the number of PDGF-BB molecules present in the system (as low as 500 fM) [206].

Analyte-dependent unfolding of aptamers can also be used to detect vascular endothelial growth factor (VEGF) [207]. Here, the analyte concentration is monitored through competitive binding of labeled complementary DNA to the analyte-specific aptamer (Figure 9). A label such as ferrocene is introduced via click chemistry, using alkyne-bearing DNA that reacts with azidomethylferrocene or other azide-containing agents. Such labeling of the DNA probe allows for a very distinct current pattern that corresponds to the probe’s translocation. With this approach, picomolar levels of VEGF can be detected [207].

Indirect sensing of volatile organic compounds has also been performed. For instance, for pesticide detection, vaporized omethoate was absorbed into an agarose gel in which it was allowed to form complexes with DNA aptamers [208]. The aptamer•omethoate complex first binds to and clogs the pore; subsequently, the applied potential leads to aptamer unfolding and translocation. Thus, long retention times are obtained for complex translocation, much longer than those for the free aptamer [208].

The applicability of nanopore biosensing for large particles was demonstrated in a study that detected *Bacillus thuringiensis* HD-73 spores with analyte-dependent aptamer unfolding [211]. Spores and aptamers were first incubated in solution and collected by centrifugation. Then, they were incubated with hairpin DNA that could further bind to the spore aptamer complex. After another centrifugation, the supernatant contained free hairpins, which were then quantified using nanopore technology [211].

## 6. Outlook

One of the long-term goals of synthetic biology is to design channel proteins *de novo*. It has been known for three decades that amphiphilic peptides can form pores or channels that are comparable in stability and ion permeability to naturally occurring channels; in addition, the amino acid composition and specific placement can be used to generate specific selectivity [219]. Compared to water-soluble proteins, there are significantly fewer reports of the synthesis of *de novo* designed membrane proteins [220]. However, significant progress has been made in both the *de novo* design [121,221] and the adaptation of existing natural structures [120]. Synthetic nanopores composed of lipid-modified DNA may also provide crucial advances toward the detection of challenging molecules [222]. An advanced understanding of the structure-function relationship of biological nanopores will enable new analytes to be tackled and innovative applications developed in the near future.

## Figures and Tables

**Table 1:** The biological pores most often employed for sensing. Representative PDB IDs are listed for oligomer structures. MW: molecular weight of the monomer, #: number, α-HL: α-hemolysin, AeL: aerolysin, MspA: *M. smegmatis* porin A, OmpG: outer membrane protein G, FhuA: ferric hydroxamate uptake component A, FraC: fragaceatoxin C, ClyA: cytolysin A, φ29p: φ29 phage DNA packaging motor, PFT: pore-forming toxin, TM: transmembrane, 1: DNA, 2: RNA, 3: nucleotide modifications, 4: peptides, 5: proteins, and 6: small molecules.

Name	Type/Organism	PDB IDs	MW (kDa)	# of Subunits	Sensing
β-barrel architecture of the TM region					
α-HL	PFT, *Staphylococcus aureus*	7AHL	34	7	1, 2, 4, 5, 6
AeL	PFT, *Aeromonas hydrophila*	5JZH	52	7	1, 3, 4, 5
MspA	porin, *Mycobacterium smegmatis*	1UUN	20	8	1
OmpG	porin, *E. coli*	2JQY	29	1	5, 6
FhuA	active transporter, *E. coli*	1BY3	79	1	5
α-helical architecture of the TM region					
FraC	PFT, *Actinia fragacea*	4TSY	20	6–8	1, 4, 5, 6
ClyA	PFT, *E. coli*, *Salmonella enterica*	6MRT^12^, 6MRU^13^, 6MRW^14^	43	8–14	1, 5
φ29p	motor pore, φ29 phage	1FOU	36	12	1, 5, 6

**Table 2:** Indirect sensing using protein pores. TBA: thrombin-binding aptamer, ABA: ATP-binding aptamer, CBA: cocaine-binding aptamer, LBA: lysozyme-binding aptamer, PDGF: platelet-derived growth factor, VEGF: vascular endothelial growth factor.

Pore	Aptamer	Analyte	Application	Reference
α-HL	G-quadruplex/TBA	monovalent and divalent metal ions	Monitoring conformational changes (folding/unfolding), examining influence of ions, *K*_d_ determination	[195,196,197,198]
	ABA	ATP, complementary DNA	Monitoring ABA conformational changes in RT upon analyte binding, *K*_d_ determination	[198,199]
	DNA hairpins		Obtaining hairpin unzipping kinetics	[200,201]
	RNA	spiropyran/merocyanine	Interaction analysis	[202]
	Pb^2+^-stabilized-DNA	lead	Detection of Pb^2+^ with a limit of 0.5 nM	[203]
	Hg^2+^-specific DNA hairpin	mercury	Detection of Hg^2+^	[204,205]
	PDGF-binding DNA	PDGF	Detection of PDGF with a limit of 500 fM	[206]
	VEGF-binding DNA	VEGF	Detection of VEGF with a limit of 5 pM	[207]
	omethoate-binding DNA	omethoate	Detection of omethoate	[208]
	CBA	cocaine	Detection of cocaine	[209,210]
	spore binding DNA hairpins	spores	Detection of *Bacillus thuringiensis* HD-73 spores	[211]
	G-quadruplex/TBA	thrombin	Probing the mechanism of TBA•thrombin binding	[96]
ClyA	TBA, LBA, hirudin	thrombin, lysozyme	Selective analyte binding and enhanced capture	[93]
FhuA	Ncp7-selected DNA aptamers	Ncp7	Measuring the affinity of Ncp7 for different DNA aptamers	[212]

## Data Availability

Not applicable.
